# Supplementary material for: A Disintegrin and Metalloproteinase with Thrombospondin Motifs 4 Regulates Pulmonary Vascular Hyperpermeability through Destruction of Glycocalyx in Acute Respiratory Distress Syndrome
Source: Int J Mol Sci. 2023 Nov 12;24(22):16230. doi: 10.3390/ijms242216230 (PMC10671186; doi:10.3390/ijms242216230)
Supplement: Supplementary file 1 [file ijms-24-16230-s001.zip › ijms-2621719-supplementary.pdf]

Table S1. Lists of genes chaged more than two-fold in microarray analysis from HMVEC-Ls stimulat

	Expression level (log2)		
Gene_Symbol	control	LPS	fold change
MYCN	9.082	5.629	-10.954
GJA4	11.898	8.703	-9.157
PDK4	9.226	6.152	-8.424
PRICKLE1	9.874	6.833	-8.231
CH25H	11.720	8.767	-7.746
RUNX1T1	11.213	8.267	-7.707
ZNF366	7.403	4.760	-6.248
DDIT4L	8.702	6.175	-5.766
HS3ST1	11.574	9.061	-5.708
FAM177B	8.943	6.457	-5.601
IL7	8.615	6.255	-5.133
HEY2	7.754	5.422	-5.035
PRICKLE2	9.007	6.682	-5.011
DDIT4L	9.887	7.619	-4.816
TRIM45	8.574	6.373	-4.601
TRIL	9.704	7.583	-4.348
PPP1R3C	9.102	6.997	-4.303
FRY	11.954	9.867	-4.247
lnc-HPDL-1	7.114	5.151	-3.900
FAXDC2	9.461	7.508	-3.872
TMEM37	9.712	7.794	-3.777
SAMD13	7.484	5.581	-3.741
CXADR	6.724	4.838	-3.698
SNRK-AS1	7.102	5.234	-3.652
PALMD	11.313	9.451	-3.636
ADGRE5	9.856	8.006	-3.606
CXCL12	11.035	9.211	-3.538
CXCR4	9.462	7.648	-3.517
FAM124B	10.602	8.802	-3.482
HOXA11-AS	7.150	5.369	-3.437
VPS37D	10.148	8.379	-3.409
CALHM2	11.518	9.751	-3.402
FREM3	10.583	8.823	-3.387
MEF2C	11.812	10.079	-3.325
ADRB1	11.254	9.530	-3.303
LONRF3	10.170	8.446	-3.303
FAM110D	9.790	8.067	-3.300
FAM124B	9.942	8.229	-3.278
TTC30B	7.594	5.893	-3.252
SMAD7	9.064	7.363	-3.251
SLC40A1	12.243	10.578	-3.170
OC10192695	7.120	5.460	-3.160
lnc-HOXA13-1	6.866	5.210	-3.151
LMCD1	9.893	8.241	-3.141
KBTBD3	6.370	4.720	-3.138
PSIP1	7.418	5.771	-3.133
NOG	11.736	10.098	-3.114
ZNF366	5.999	4.368	-3.099
CYP1A1	10.023	8.395	-3.091
FOXD1	6.875	5.253	-3.078
ACKR4	7.539	5.920	-3.073
ARHGAP28	6.740	5.121	-3.072
HOXA3	8.641	7.025	-3.066

	Expression
Gene_Symbol	control
DDIT4L	9.887
MYCN	9.082
DDIT4L	8.702
CXCR4	9.462
PPP1R3C	9.102
lnc-TSC22D1-1	7.796
PDK4	9.226
GJA4	11.898
HEY2	7.754
RAVER1	12.767
LINC00271	10.001
PRICKLE1	9.874
ADRB1	11.254
TRIM45	8.574
IQSEC3	8.861
RUNX1T1	11.213
HCN2	13.422
LCE1A	12.602
PRRT3-AS1	12.123
FAM124B	10.602
RNF165	13.788
LOC10536958	14.386
LOC10012991	9.703
	13.581
PLCH1-AS2	10.576
VPS9D1	15.110
PPDPF	13.192
LOC10536997	10.028
STIM2	6.961
MON1B	12.237
lnc-VWF-2	14.703
PPIEL	6.966
HS3ST1	11.574
CISD3	11.591
DUSP4	9.201
UTS2R	12.425
LOC10272396	9.545
C1orf229	10.694
PRICKLE2	9.007
FAXDC2	9.461
VPS37D	10.148
GPR150	14.186
PLD6	7.390
LOC10798420	12.141
FAM124B	9.942
CD72	13.497
	13.448
	14.959
WEE2-AS1	6.396
NUAK1	14.536
	14.114
VASH1	12.068
ZNF30	8.350

ZNF395	10.341	8.725	-3.066	LCE1C	11.859
HOXA10	6.806	5.192	-3.059	SYNGR4	7.134
TMC7	8.441	6.832	-3.052	CALY	13.959
lnc-CDK17-1	7.100	5.492	-3.048	CXCL12	11.035
EHD3	8.796	7.190	-3.043	LMCD1	9.893
TMCC3	8.606	7.005	-3.035	HGC6.3	10.958
CRYAB	9.085	7.485	-3.030	FAM27E2	8.806
NUAK1	14.536	12.945	-3.013	lnc-HPDL-1	7.114
ARHGAP5-AS1	6.238	4.652	-3.002	KBTBD3	6.370
TBX1	13.355	11.769	-3.001	FAM110D	9.790
GATA3	6.576	4.994	-2.995	TPTE2	6.793
ZSWIM5	10.436	8.854	-2.993	HOXD1	10.703
SPAAR	9.984	8.411	-2.975	lnc-RPS4XP21-	6.729
BCL11A	6.244	4.678	-2.960	SMIM35	10.422
KAZALD1	7.640	6.088	-2.933	GFOD1	11.889
DUSP4	9.201	7.654	-2.923	TMCC3	8.606
FAM78A	7.918	6.377	-2.910	DNAJC9-AS1	7.038
CXCL12	10.355	8.818	-2.901	BCL11A	6.244
IRF6	7.697	6.168	-2.886	SAMD13	7.484
CNTNAP3B	6.538	5.019	-2.867	LONRF3	10.170
lnc-DCAF10-2	8.460	6.942	-2.865	PPARGC1B	7.346
NEXN	9.532	8.027	-2.838	EN2	13.105
nc-RPS4XP21-	6.729	5.225	-2.837	LYNX1-SLURP1	9.865
FZD1	10.786	9.286	-2.830	SEC14L2	7.210
	7.054	5.561	-2.815	FRY	11.954
	6.204	4.712	-2.814		12.820
ZNF30	7.695	6.203	-2.812	ARL4A	9.890
MMP16	6.707	5.217	-2.809	lnc-HOXA13-1	6.866
IL21R	7.321	5.834	-2.802	SCGB3A1	13.345
ARHGEF37	10.156	8.676	-2.788	RAB11FIP1	8.069
GPAT3	8.032	6.564	-2.766	TRIL	9.704
RGCC	13.829	12.362	-2.765	TMEM238	12.698
MAFB	10.287	8.828	-2.749	ARL4A	11.121
lnc-OSBPL10-1	6.869	5.415	-2.740	lnc-DCAF10-2	8.460
RAB11FIP1	8.069	6.616	-2.737	ACACB	6.580
FLJ37035	7.523	6.083	-2.713	SSR4P1	8.186
DENND2C	9.009	7.580	-2.692	FZD1	10.786
RAET1E-AS1	7.532	6.103	-2.692	NOG	11.736
TLE1	12.055	10.627	-2.691		6.973
LOC10027074(	6.496	5.069	-2.690	LOC10012909(	7.228
SYBU	7.165	5.741	-2.685	SPAAR	9.984
GFOD1	11.889	10.470	-2.674	IL7	8.615
USP27X-AS1	6.821	5.405	-2.669	TBX1	13.355
ZNF30	8.350	6.939	-2.659	BCL9	7.865
SMAD6	14.332	12.926	-2.650	NKX2-5	14.766
IGFBP5	7.654	6.251	-2.644	TSC22D3	10.555
LOC_I2_00953	7.779	6.380	-2.637	ZNF30	7.695
PNMA8A	7.095	5.697	-2.636		16.711
MARC1	7.059	5.663	-2.632	JADE1	10.163
EPB41L4B	6.736	5.341	-2.629	RTL10	10.747
TMEM121	10.837	9.449	-2.616	ZMYND8	6.227
HOXB6	13.140	11.754	-2.613	LOC10798520(	7.287
SOX13	7.758	6.379	-2.602	ZNF792	7.869
	6.093	4.720	-2.591	SNORA71A	9.080
LINC01693	7.369	5.999	-2.584	HOXA11-AS	7.150
	6.973	5.605	-2.581	RNF152	9.354
lnc-TSC22D1-1	7.796	6.428	-2.580	LIPT2	7.847

MAFB	12.841	11.475	-2.578	TUBA3D	7.869
HOXD1	10.703	9.337	-2.576	SNORA71A	6.210
RNF152	9.354	7.991	-2.572	FRAT1	7.937
FOXS1	6.262	4.899	-2.571	INKA2	8.975
lnc-ARL13A-1	6.075	4.715	-2.567	GPAM	6.340
HOXA11-AS	8.692	7.333	-2.566	TLE1	12.055
SSR4P1	8.186	6.827	-2.564	TMEM37	9.712
	6.429	5.074	-2.559	TOR2A	12.447
WEE2-AS1	6.396	5.044	-2.553	GALR3	12.716
HOXA2	7.172	5.822	-2.549	lnc-CDK17-1	7.100
ETV1	6.994	5.648	-2.542	RAET1E-AS1	7.532
LSM11	5.860	4.516	-2.537	PXMP4	7.999
LOC10798449	5.989	4.646	-2.536	PDE7B	8.508
	6.250	4.912	-2.527	TNRC18	14.334
FAM84B	7.552	6.220	-2.518		6.602
SEMA3G	10.824	9.494	-2.514	LINC01091	9.660
MBOAT1	9.532	8.203	-2.512	SMAD6	14.332
ZNF792	7.869	6.547	-2.501	FAM217B	10.913
TMSB4X	14.552	13.240	-2.484	SMAD7	9.064
LOC10192830	7.037	5.728	-2.477	lnc-OPN4-2	11.405
LNX1	6.279	4.973	-2.473	HOXA9	11.236
REEP1	8.674	7.373	-2.464	PPA2	11.071
ZNF521	9.144	7.846	-2.460		14.403
LOC10798520	7.287	5.992	-2.454	TMEM121	10.837
	6.254	4.959	-2.454		11.484
STIM2	6.961	5.667	-2.452	CCM2L	14.047
FRMD3	10.151	8.858	-2.450		9.027
PPARGC1B	7.346	6.054	-2.450	CD79A	10.560
FRMD3	9.709	8.420	-2.444	NLRC3	12.429
	5.724	4.437	-2.440	lnc-KIF25-2	8.829
CXCL12	11.825	10.541	-2.437	GATA3	6.576
C6orf141	6.154	4.870	-2.436		11.228
SLC1A1	10.024	8.743	-2.430	TUSC8	13.815
ELMOD1	7.516	6.237	-2.428		15.163
LIMD1-AS1	5.902	4.623	-2.427	FGD4	10.217
NR3C2	8.815	7.539	-2.421	ARHGEF37	10.156
ARHGEF3	8.216	6.941	-2.420	DACT1	6.775
CMTM8	11.623	10.348	-2.420	LINC01869	10.674
CAVIN2	11.265	9.992	-2.417	CRYAB	9.085
PIK3CG	7.154	5.882	-2.415	CXCL12	10.355
LOC283177	6.089	4.825	-2.400	DNM1	15.539
KANK2	12.235	10.972	-2.399	SMPD5	11.917
RIMS1	10.079	8.819	-2.393	E2F2	8.590
DACT1	6.775	5.520	-2.388	LINC01091	9.881
NLRC3	7.856	6.604	-2.382	SLC40A1	12.243
PLD6	7.390	6.141	-2.378	CNTNAP3B	6.538
ANXA2R	11.899	10.654	-2.370	ADGRE5	9.856
LOC10192934	7.594	6.351	-2.366		11.275
ZCCHC2	6.690	5.450	-2.362	lnc-TMEM99-5	8.700
FAT4	8.445	7.211	-2.352	lnc-OSBPL10-1	6.869
	7.037	5.805	-2.349	SNORA71B	6.184
LOC10192830	7.442	6.217	-2.339	PNMA8A	7.095
FAM198B-AS1	6.460	5.235	-2.337	LNX1	6.279
FAM214A	10.954	9.730	-2.336	ZNF552	6.562
	6.042	4.820	-2.332	SLC12A5	9.410
DLG3	6.614	5.393	-2.330	CALHM2	11.518
ZNF594	5.771	4.554	-2.325		14.716

HECW2-AS1	6.344	5.127	-2.324	lnc-DOLPP1-1	9.995
KLLN	6.246	5.030	-2.322	CASKIN1	12.941
SLCO2B1	6.696	5.482	-2.319	FAM50B	10.235
LOC153684	7.243	6.031	-2.315		7.161
PDE7B	8.508	7.299	-2.312	ZSWIM5	10.436
FRAT1	7.937	6.728	-2.311	CYREN	9.519
THEM6	11.997	10.791	-2.308	FAM78A	7.918
ZNF658	9.356	8.150	-2.308	FREM3	10.583
ABCC6	5.800	4.595	-2.305	ZNF34	6.187
KLRG1	5.546	4.343	-2.302		10.729
ZNF382	5.726	4.525	-2.300	LOC10013328(	7.479
	6.602	5.401	-2.299	LOC728613	7.743
lnc-COG3-2	7.283	6.087	-2.292	lnc-CTTN-3	7.920
TRIM46	7.355	6.158	-2.292	LYL1	9.714
FRRS1	7.531	6.337	-2.289		6.254
RAMP3	7.526	6.334	-2.285	FOXS1	6.262
PRAG1	13.366	12.175	-2.283	C6orf141	6.154
FGD4	10.217	9.028	-2.279	PALMD	11.313
VANGL1	12.167	10.980	-2.277	lnc-BEGAIN-1	15.946
TLE1	8.204	7.017	-2.277	KANK2	12.235
	6.395	5.208	-2.277		14.348
TCAF1	8.778	7.593	-2.274	ZNF395	10.341
ANKMY2	6.650	5.465	-2.274	TRIM46	7.355
RPRD2	9.841	8.656	-2.273	TMC7	8.441
ZNF503	9.892	8.707	-2.273	KBTBD7	7.474
LINC00847	6.811	5.633	-2.263		13.979
LOC10537632!	8.499	7.322	-2.261	TMEM246	13.274
STARD9	6.180	5.004	-2.258	FOXB1	8.275
ZNF575	8.089	6.914	-2.257	GPAT3	8.032
TMEM246	13.274	12.104	-2.249		16.018
ABHD6	10.009	8.840	-2.248	ZNF658	9.356
HOXA9	11.236	10.074	-2.238	SNORA71C	6.365
ACACB	6.580	5.423	-2.231	OSBPL7	11.365
SKIDA1	6.420	5.264	-2.229	lnc-FBLN2-1	9.513
SATB1	8.925	7.769	-2.228	LINC00923	7.045
EBF3	7.162	6.006	-2.228		12.160
LRRC70	11.315	10.159	-2.227	PPARGC1B	6.238
LOC10012909{	7.228	6.075	-2.224	ACTL10	8.056
C19orf57	5.660	4.506	-2.224	PCDH9	7.794
C14orf132	10.875	9.725	-2.219	LINC01410	6.373
ZCCHC2	10.249	9.100	-2.217	TIGD2	8.470
FLJ45513	7.209	6.060	-2.217	FAM27E3	6.375
lnc-TMEM99-5	8.700	7.554	-2.213	LRRC70	11.315
MFAP3L	7.536	6.389	-2.213		6.250
CYFIP2	6.210	5.065	-2.211		10.665
LOC153684	8.105	6.961	-2.210	DENND2C	9.009
SRRM3	9.118	7.975	-2.209	STARD9	6.180
SMARCA2	7.879	6.736	-2.209	HOXA11-AS	8.692
ZKSCAN4	6.336	5.198	-2.202	NLGN2	12.571
ZFP64	9.549	8.411	-2.200	lnc-MTERFD3-:	6.368
GNRHR2	6.195	5.058	-2.199	TSHZ1	10.291
lnc-C5orf42-2	5.590	4.459	-2.191	GLI4	13.537
	8.492	7.361	-2.190	HOXB6	13.140
RARA	10.608	9.480	-2.186	FZD8	8.572
SLC26A4	7.400	6.272	-2.185	LFNG	11.062
FAM50B	10.235	9.108	-2.184	FRMD3	9.709
MYZAP	6.821	5.697	-2.180	ZNF284	9.118

ZNF521	12.124	11.002	-2.177	USP35	10.727
ZHX3	7.325	6.205	-2.173	LINC01089	10.390
FAT4	7.677	6.559	-2.172	PRAG1	13.366
GPAM	6.340	5.221	-2.172	LINC01001	12.392
DMAC1	6.304	5.186	-2.170	AJUBA	9.632
EFCC1	8.432	7.317	-2.166		7.701
ORAI3	7.274	6.160	-2.164	RARA-AS1	6.668
FLJ32255	9.020	7.906	-2.164	LOC10050593‹	10.228
LOC729080	8.142	7.029	-2.163	LOC10798538‹	14.638
CERS6-AS1	7.215	6.102	-2.163	PROX1	8.399
PELI2	7.722	6.611	-2.159	SPRY4-IT1	9.423
PCDH9	7.794	6.684	-2.159		12.428
LPAR6	10.278	9.168	-2.158	BCAR1	8.234
LOC257396	6.459	5.350	-2.157	MYLIP	9.389
TSC22D3	10.555	9.447	-2.155		12.124
THAP8	7.222	6.114	-2.155	ZNF329	8.909
PCSK9	5.983	4.876	-2.154	ZKSCAN7	7.290
PXMP4	7.999	6.897	-2.147	TTC30B	7.594
HOXD9	9.233	8.131	-2.146	MESP1	7.926
	6.280	5.181	-2.142		7.037
JADE1	10.163	9.065	-2.141	BMP4	9.820
_OC10192798‹	5.850	4.753	-2.140	SYBU	7.165
LINC00847	10.232	9.136	-2.138	CXCL12	11.825
ENC1	9.341	8.247	-2.135	lnc-EVX1-5	7.959
HOXD8	8.845	7.753	-2.131		6.624
ZNF552	6.562	5.471	-2.130	RGCC	13.829
	5.678	4.587	-2.130	RARA	10.608
IRAK1BP1	6.588	5.499	-2.128	ZADH2	9.211
SLC2A12	6.150	5.064	-2.122	FAM53B	10.707
LINC01091	9.881	8.797	-2.121	LOC10537632‹	8.499
FAM84A	6.296	5.211	-2.121	TIRAP	5.819
IER5L	9.174	8.090	-2.120	TPP1	12.615
AP3M2	9.690	8.608	-2.118	FAM117B	8.946
FAM53B	10.707	9.626	-2.115	PPP1R3E	5.990
GIMAP1	11.819	10.739	-2.115	CHST6	11.247
	5.707	4.628	-2.112	NR2F2	14.066
GPT2	8.657	7.579	-2.112	SNHG20	9.269
MYZAP	10.929	9.851	-2.111	LINC02188	5.956
CEP68	6.792	5.715	-2.110	CYP1A1	10.023
CCDC121	7.088	6.011	-2.109	RAB30	7.366
SSSCA1-AS1	5.957	4.886	-2.101	LOC10192830‹	7.037
RFX2	10.112	9.041	-2.101	SLCO2B1	6.696
ADAMTS1	7.682	6.612	-2.100	lnc-ARL13A-1	6.075
KCNJ18	6.588	5.520	-2.097		9.036
RNF182	9.015	7.947	-2.096	CRTC1	8.206
FLJ32255	8.983	7.919	-2.091	C21orf58	7.462
ZBTB16	8.784	7.720	-2.090	RIN1	10.073
ZNF174	5.677	4.614	-2.090	ZADH2	10.667
DNAJC9-AS1	7.038	5.975	-2.088	LINC01747	5.838
MBLAC2	9.066	8.004	-2.087	RAPH1	10.868
FAM199X	7.631	6.570	-2.087	DIABLO	14.937
	6.997	5.937	-2.086	WWC2-AS2	7.092
SMIM10L2B	8.195	7.140	-2.078	PRR36	12.098
MOB3B	7.150	6.095	-2.078	PLEKHG3	9.608
lnc-C11orf1-1	6.440	5.385	-2.077		7.186
LBH	6.303	5.249	-2.076	LOC10537628‹	6.564
LINC01006	7.043	5.990	-2.075	NALT1	6.884

HTR2B	8.317	7.264	-2.075	FOXD1	6.875
NLRP12	5.933	4.881	-2.074	ELFN1	11.784
lnc-LEO1-2	8.640	7.589	-2.072	PLEKHH3	9.700
RIN1	10.073	9.023	-2.070	CEP68	6.792
STON1	9.545	8.495	-2.070	FAM177B	8.943
LIPT2	7.847	6.799	-2.067	LINC00847	10.232
ETFRF1	6.241	5.194	-2.066	FOXP4	12.713
LINC00640	6.625	5.579	-2.065		9.548
	6.048	5.003	-2.063	PCSK4	6.262
NR2F2	14.066	13.023	-2.061	ZSCAN16	6.827
TRIM6	7.323	6.280	-2.061	ARHGAP33	13.165
NIPSNAP3B	5.412	4.371	-2.058		9.411
ZNF658	7.442	6.401	-2.057	LOC10192695(	7.120
ANKRD20A12F	5.570	4.531	-2.055	HOXA9	9.299
LXN	13.316	12.277	-2.054	lnc-TPSAB1-1	16.425
WWC2-AS2	7.092	6.055	-2.052	KRTAP19-8	6.035
lnc-PELI2-5	5.579	4.546	-2.045	GDPGP1	6.149
lnc-MYOF-1	6.303	5.271	-2.045	TMEM223	11.444
GPRASP1	6.904	5.873	-2.044	MINCR	6.003
SMIM10L2B	6.389	5.358	-2.043	EWSAT1	6.927
ZNF571	6.565	5.534	-2.043	ZBTB16	8.784
TMEM223	11.444	10.414	-2.042		6.429
TAOK3	7.768	6.741	-2.039	SH2D3C	13.268
C10orf142	5.883	4.857	-2.038	SNRK-AS1	7.102
MITF	7.493	6.467	-2.037	DGKZ	6.013
MFSD6	7.864	6.838	-2.035	HOXA9	7.677
ARL4A	9.890	8.865	-2.034		7.054
INKA2	8.975	7.951	-2.034	FAM84A	6.296
FAM92A1P2	5.986	4.962	-2.034	IER5L	9.174
LURAP1	5.417	4.396	-2.030	FLJ42351	6.376
MYLK4	6.198	5.177	-2.029	SPSB2	11.028
DNMBP	11.051	10.030	-2.029		6.280
ADAP2	6.795	5.775	-2.028	PGF	14.535
SEMA4D	5.613	4.594	-2.026	LINC00685	7.155
TRIB2	6.644	5.627	-2.024	SEMA3G	10.824
TMEM170B	6.409	5.392	-2.024	ZNF331	8.362
PROX1	8.399	7.382	-2.024	ADAT2	7.035
LMX1B	6.500	5.484	-2.022	SC5D	5.845
ATOH8	10.519	9.505	-2.019	FAM78B	5.855
FAM117A	12.041	11.028	-2.019	LINC02138	13.034
SSUH2	7.318	6.306	-2.017	NEXN	9.532
SLC38A3	7.979	6.967	-2.017	NOL4L	7.261
LINC01091	9.660	8.648	-2.016	CH25H	11.720
LMO4	10.278	9.267	-2.015	SFTA1P	13.864
AJUBA	9.632	8.623	-2.013		8.933
	5.863	4.854	-2.013	LINC02593	6.758
KLHL23	6.244	5.236	-2.012	ZFP30	9.715
SOCS2-AS1	7.511	6.503	-2.011	FRMD3	5.787
	7.509	6.502	-2.010		6.337
GNAI1	9.365	8.359	-2.009	ZNF436-AS1	9.042
PPP1R14A	7.835	6.828	-2.009	MELTF-AS1	8.192
	8.589	7.584	-2.007	C14orf132	10.875
LOC728392	10.613	9.609	-2.006	THEM6	11.997
FAM117B	8.946	7.942	-2.005	FLJ37035	7.523
BCORL1	8.775	7.772	-2.005		14.255
REPS2	6.188	5.185	-2.005	LOC10537033	10.137
ASB13	10.452	9.449	-2.004	DUX4	15.405

CHST15	10.902	9.899	-2.004	CIRBP	8.841
BCL11A	7.969	6.967	-2.004	EXOC3L2	7.349
ZKSCAN3	5.476	4.475	-2.002	NCK1-DT	6.848
FBH1	5.832	6.832	2.001	C20orf204	9.640
RPL23AP32	6.636	7.637	2.002	SFXN5	8.176
SVIL2P	5.518	6.520	2.002	C21orf58	6.650
LOC114224	4.840	5.842	2.003	MFAP3L	7.536
MEG3	5.170	6.173	2.004	MMP16	6.707
OGFR	10.042	11.046	2.005	lnc-TMEM106C-	12.198
BACH1	10.449	11.453	2.006	SNORD11	6.005
HDX	7.532	8.537	2.007	TLE1	8.204
SELENOM	10.961	11.966	2.007	PPP1R14A	12.562
EGR2	4.671	5.676	2.007	PDIK1L	7.529
CEMIP	5.826	6.832	2.009	CDK5R1	8.195
TAB2	12.362	13.371	2.012	ARAFP2	14.429
COL27A1	5.499	6.508	2.012	PDP2	10.302
FGF2	7.242	8.252	2.015	COX11	6.328
CD274	8.603	9.614	2.015	HOXA2	7.172
STARD10	8.196	9.208	2.016	HEXIM2	10.626
ARF3	4.823	5.835	2.017	NR5A2	8.289
SAMD9L	8.310	9.322	2.017		7.509
CHIC2	10.548	11.561	2.019	GS1-278J22.2	10.539
ATAD3C	7.387	8.404	2.023	TPSG1	15.006
AKT2	5.230	6.247	2.024	ZNF671	8.640
LINC02085	6.843	7.860	2.025	ZSCAN12	6.689
ADPRHL2	9.534	10.553	2.026	RIMS1	10.079
CLIP2	9.937	10.957	2.027	PGBD3	8.666
CSGALNACT2	4.833	5.853	2.028	MINCR	8.312
GLYCTK	4.798	5.819	2.029	HOXA9	9.836
lnc-GTF2F2-2	5.064	6.085	2.030	LOC10798709{	5.796
lnc-IRX3-4	4.470	5.492	2.030	IRAK1BP1	6.588
LYRM1	9.155	10.177	2.031	GDPGP1	6.989
FRMD8	5.035	6.058	2.031	GPX8	13.367
CTSL	7.559	8.582	2.033	ANKRD23	6.250
PKIG	10.739	11.763	2.034	EPB41L4B	6.736
RELT	10.798	11.824	2.036	CAVIN2	11.265
BCL2L11	10.826	11.852	2.036		6.204
lnc-CETP-1	7.500	8.526	2.037	LINC01693	7.369
SNORD114-21	7.612	8.640	2.038	FLJ20021	9.865
CAB39	10.558	11.586	2.040	BRICD5	7.638
TRMT44	4.901	5.931	2.042	DUXAP9	8.952
NANP	5.723	6.754	2.043	CHST15	10.902
GPR37L1	6.251	7.282	2.043	HOXA3	8.641
PRDM8	7.702	8.734	2.044	ZNF397	6.799
CSGALNACT2	8.194	9.227	2.046		5.863
EVA1C	8.375	9.409	2.048	CCDC9B	11.925
JAK3	10.360	11.394	2.048	ADRB2	10.673
S1PR1	4.987	6.021	2.049	LOC10192934(	7.594
EDC4	5.730	6.766	2.051	MID1IP1	10.749
MEG9	7.103	8.140	2.051	LSM11	5.860
HOXD10	9.465	10.503	2.054	EHD3	8.796
ADAP1	5.980	7.019	2.055	KLLN	6.246
TRIM21	7.186	8.226	2.056	lnc-RP11-422N16.3	6.609
RPS21	5.629	6.669	2.056	MARC1	7.059
KIF21B	7.360	8.400	2.056	ENC1	9.341
TLCD1	6.978	8.019	2.059	lnc-CCDC90A-5	9.623
NFKBIE	12.531	13.573	2.059	ZNF594	5.771

SLC7A11	13.141	14.184	2.061	XLOC_l2_01187	5.580
LOC_l2_00486	6.753	7.796	2.061	UQCC1	8.602
	5.480	6.524	2.061	TMEM170B	6.409
UBE2Z	7.914	8.958	2.061	PSMG3-AS1	12.555
ELMO3	5.350	6.397	2.066	RIBC2	5.805
BCL6B	10.970	12.017	2.066		5.411
XPO6	5.244	6.293	2.068	ATOH8	10.519
EHMT1	6.629	7.678	2.069		8.215
CHMP1A	7.014	8.064	2.070	IQGAP2	10.549
QSOX1	7.269	8.320	2.071	lnc-C11orf1-1	6.440
HOXB9	5.880	6.933	2.075	ZFP2	6.321
PSTPIP2	5.766	6.819	2.076	ZFP64	9.549
PNPLA8	10.760	11.814	2.076	PLEKHG3	9.922
	4.463	5.517	2.077	TMEM177	11.305
SHISA3	6.774	7.829	2.078	MBLAC2	9.066
LOC728715	7.333	8.388	2.078	MST1	8.556
	5.938	6.994	2.079	LOC728392	10.613
COL27A1	11.346	12.403	2.079	ENGASE	8.859
	5.065	6.123	2.083	LRRC20	10.312
CFLAR	10.146	11.205	2.084	CMTM8	11.623
NAV2	6.772	7.833	2.086	CLEC3B	7.524
BBC3	12.853	13.917	2.090	XLOC_l2_00756	6.305
SNORD114-15	7.058	8.123	2.093	ORAI3	7.274
USP12	5.585	6.651	2.094	CDH22	10.629
PDE4DIP	6.982	8.049	2.095	lnc-FBXO25-3	6.544
GPAT4	9.235	10.303	2.097	KAT14	11.291
SULT1C4	5.195	6.263	2.097		8.571
TGIF1	9.430	10.500	2.099	TTLL8	9.162
LONRF1	5.493	6.563	2.100	SGO1-AS1	5.947
MICALL1	8.028	9.100	2.102	RFX2	10.112
SLC12A2	10.501	11.575	2.105	MYZAP	10.929
FERMT2	8.884	9.959	2.106	ZNF681	5.894
ZNF697	8.337	9.411	2.106		8.843
GOLGA8A	4.693	5.768	2.107	lnc-DHX15-1	10.040
CD40	8.841	9.916	2.107	NFKBIL1	13.481
ZSCAN22	5.017	6.094	2.109	CBX2	10.940
SAV1	11.990	13.067	2.110		5.420
DENND4A	5.157	6.234	2.110	lnc-RP11-712L6.5	9.148
SERPINE1	12.318	13.397	2.112	SNORA80B	7.226
AP5Z1	7.094	8.173	2.113	HMCN1	6.885
lnc-CETP-1	5.979	7.058	2.113	NLRC3	7.856
KDM2A	8.889	9.969	2.115	LINC00847	6.811
SAMD9L	10.482	11.564	2.117	ADARB2-AS1	11.269
TTC39A	5.682	6.765	2.119	ZNF215	7.941
TBL3	7.582	8.665	2.119	HOXA10	9.501
RPS4X	5.683	6.767	2.120	ZKSCAN4	6.336
DNAJB5	8.229	9.313	2.121	LINC00537	11.924
RBM23	4.910	5.997	2.123	LOC257396	6.459
FAM126A	5.443	6.531	2.125	ZNF571	6.565
TBILA	6.715	7.803	2.126	PTMS	15.612
VEGFA	5.229	6.318	2.127	SMR3A	5.458
LINC00964	7.061	8.158	2.139	ZNF551	7.263
lnc-MFSD9-4	5.936	7.035	2.142	USP27X-AS1	6.821
EVA1C	8.731	9.833	2.148	NAGS	9.876
HIPK2	6.185	7.290	2.151	VMAC	7.118
YPEL2	10.304	11.412	2.156	IL21R	7.321
MAP2K3	7.437	8.546	2.157	RNF113B	5.573

NDEL1	7.162	8.272	2.158	PTCH1	5.676
HCP5	5.213	6.323	2.159	POC5	8.287
CTHRC1	12.592	13.703	2.160	lnc-RNASEH1-2	6.034
C17orf107	7.758	8.870	2.163	MAP4	16.075
MICAL1	5.299	6.413	2.165		5.714
nc-C16orf95.1-	5.561	6.675	2.165	lnc-C21orf58-1	6.559
GCA	7.079	8.194	2.165	MOCS3	12.032
LOC440028	4.818	5.933	2.167		6.229
C17orf53	5.484	6.600	2.167		6.151
SLC31A2	10.515	11.632	2.169	ZNF850	6.978
KLF3	11.487	12.605	2.171	CCDC78	10.622
PLK3	12.145	13.264	2.172	LBX1	14.541
SNORD114-5	5.988	7.108	2.174	LOC283140	10.532
ZNF71	8.462	9.582	2.174	HLX	8.468
GMEB2	6.527	7.647	2.174		9.848
PCDH1	5.890	7.011	2.176		8.861
SMG5	6.194	7.317	2.177	LHX3	10.077
CDC42EP2	11.324	12.447	2.179	CEP19	6.379
TMEM135	4.609	5.735	2.183	LOC10050562	5.078
LYPD6	4.691	5.819	2.184	LY6E	9.948
MCTP1	9.363	10.492	2.187	VGLL4	8.822
PANX1	11.041	12.170	2.187	BRD9	6.614
DDX3Y	5.422	6.553	2.191	IER2	14.944
HSPA8	4.368	5.505	2.200	GABRE	9.719
KDM7A	5.064	6.202	2.201	VEGFC	13.087
SERPINE2	9.828	10.967	2.201	EVA1C	8.375
DNM3OS	6.512	7.652	2.205	GIMAP8	11.686
CBLN3	5.393	6.538	2.211	SNAR-A3	16.815
B3GNT5	11.189	12.335	2.212	MAP2K3	7.437
BIRC2	11.342	12.488	2.214	XLOC_l2_00569	10.131
LINC00240	5.112	6.261	2.218	SLC18A3	6.787
SNORD114-11	4.614	5.764	2.219	RIN2	12.088
RGS3	12.021	13.173	2.222	SMIM17	5.513
RUNX1	9.353	10.507	2.225	FAM124A	8.075
NFKBIB	8.608	9.763	2.227	CNP	15.097
	6.387	7.542	2.228	PLAUR	9.795
HTATSF1P2	5.869	7.026	2.230	RNF145	5.822
DNAJB9	12.662	13.821	2.232	KCTD5	6.404
PML	6.503	7.662	2.233	LINC02190	5.138
MAST4	11.100	12.261	2.236	SNAR-D	15.915
LOC646626	9.236	10.397	2.236	LZTS3	5.620
	6.784	7.945	2.237	PDE1C	5.383
IL32	11.887	13.049	2.239	lnc-GTF2F2-2	5.064
SMIM17	5.513	6.677	2.241	LARP6	9.502
ZFPM2	10.196	11.360	2.241	DCAF6	5.543
ZDHHC11	4.612	5.777	2.242	PDGFRA	5.095
PPP2R5B	6.249	7.414	2.243	CTHRC1	12.592
HEY1	11.785	12.951	2.244	ODF3B	6.809
TICAM1	12.969	14.138	2.248	TTC39C	5.123
FZD9	7.227	8.396	2.248	KLF10	10.103
CYP1A2	5.459	6.628	2.248	SERPINE2	9.828
ANKLE2	8.374	9.545	2.251	PATL1	8.866
ATAD3B	8.163	9.334	2.251	TLE4	6.959
KDM6B	5.535	6.708	2.255	GPR68	9.083
PARP14	9.579	10.753	2.256	TRIM5	7.317
lnc-ID1-1	5.355	6.534	2.265	HNRNPU	4.931
LAMC2	10.982	12.163	2.268	ATXN7	4.607

SCARNA7	7.367	8.549	2.269	RBM43	7.919
MAPK8	5.244	6.428	2.272	LOC114224	4.840
ZNFX1	5.147	6.331	2.273	BTN2A2	8.798
lnc-CRISP2-1	4.829	6.015	2.275	KDM7A-DT	5.598
OR10G2	5.007	6.194	2.277	CYB5R2	7.207
ZSWIM4	8.766	9.956	2.281		8.289
	5.099	6.289	2.281	XXYLT1-AS2	6.621
KLF9	11.167	12.357	2.282	GPBP1	11.243
	8.653	9.844	2.284	DNPEP	7.224
SLC25A37	9.205	10.397	2.285	IKBKE	6.088
	6.542	7.735	2.286	SELENOM	10.961
SNORD114-22	6.628	7.821	2.286	ZBTB43	7.214
LOC_l2_00569	10.131	11.325	2.288	CHST6	6.128
BCL9L	7.412	8.607	2.289	XLOC_l2_00329	8.234
PALLD	11.614	12.809	2.290	OVOS2	9.459
PLAU	13.138	14.334	2.291	MEAK7	5.118
SLC15A3	7.448	8.650	2.302	F2RL1	10.771
IER5	13.331	14.535	2.303	NMI	11.208
SUSD6	11.720	12.929	2.312	ANKMY1	6.318
LARP6	9.502	10.715	2.319	SLC18B1	5.358
CCNL1	8.301	9.516	2.321	HIPK1	4.805
TNFAIP1	13.551	14.766	2.321	CHAC1	9.038
DRAM1	12.915	14.131	2.323	SP140	5.301
GJD3	9.242	10.460	2.326	SLFN12	8.282
ELL2	10.559	11.779	2.328	SNAR-G2	15.058
NAB1	9.391	10.611	2.331	HAPLN3	13.847
TANK	10.696	11.918	2.333	RELA	8.777
SAT1	13.686	14.911	2.337	PNPLA8	10.760
MFSD2A	7.948	9.174	2.339	MAST4	11.100
ABHD17B	5.528	6.754	2.339	ZNF428	14.343
CDKN1A	8.573	9.799	2.340	OSMR	5.180
STC2	6.301	7.532	2.347	KBTBD2	9.083
CYLD	8.064	9.297	2.350	TGIF1	9.430
S1PR1	11.740	12.974	2.353	LOC10192796З	4.634
ISG20	12.929	14.164	2.354	CD40	8.841
MMP25	4.509	5.745	2.355	DNAJB5	11.680
ZNF217	8.617	9.854	2.357		4.848
HIF1A	7.339	8.578	2.361	UBE2Z	7.914
NR3C1	4.768	6.008	2.362	RABGAP1L	5.995
CCNT1	5.157	6.399	2.364	RBBP6	7.017
STAT5A	7.991	9.233	2.365	EHD1	12.763
TMEM171	8.400	9.642	2.366	ZHX2	7.614
DPF3	6.646	7.891	2.371	DNAJC27-AS1	7.140
	4.892	6.143	2.379	RUNX1	9.353
TNFSF18	5.652	6.904	2.382	NUB1	9.775
lnc-RELT-1	5.472	6.725	2.384	RHOB	13.257
BHLHE40	8.042	9.298	2.388	PRKCD	8.824
ZNFX1	8.186	9.445	2.393	LAMC2	10.982
LACC1	6.487	7.749	2.399	KDM6B	5.535
ARID3B	9.370	10.635	2.403	SNORD3B-1	11.297
RBM20	6.941	8.211	2.413	CPEB2	5.830
APOL1	9.756	11.027	2.413	PSMD6-AS2	5.467
BCOR	5.539	6.812	2.417		5.373
APOBEC3G	6.767	8.041	2.418	SNAR-B2	16.288
IL32	10.724	12.000	2.421	B4GALT5	11.909
TAP1	12.327	13.604	2.425	lnc-RP11-127H5.1	4.775
	7.429	8.710	2.429	LOC10013283	9.533

CCDC80	4.647	5.928	2.430	CA13	8.114
PELI1	11.848	13.130	2.433	NIPA1	11.054
MAML2	5.998	7.282	2.435	TLE4	9.564
SMAD3	11.539	12.823	2.435	PARP10	10.815
CHAC1	9.038	10.324	2.438	IER5	13.331
CD40	7.671	8.957	2.439	SLC41A1	10.555
NR1D1	4.763	6.050	2.440	AADACL3	6.041
CCNL1	8.608	9.895	2.441	KREMEN1	5.833
RNF145	5.822	7.110	2.442	CHIC2	10.548
SH3RF3-AS1	6.701	7.989	2.442	CAB39	7.919
CLUHP3	4.715	6.004	2.443	ZNF267	8.907
BMP2	10.643	11.933	2.445	PPP1R15A	9.838
FAM178B	7.687	8.977	2.445		9.017
PLLP	5.284	6.574	2.446	MYD88	10.826
GPR68	9.083	10.380	2.456	LINC01629	4.980
GEM	5.019	6.320	2.463	BNC2	7.451
WTAP	11.698	13.000	2.466	LAMB3	9.555
ACHE	4.977	6.283	2.473	GNAI3	4.950
STARD13	4.734	6.041	2.473	LOC11226817(	5.697
RC3H1	10.443	11.751	2.475	SCYL2	4.761
MYOM1	5.255	6.564	2.479	ASPHD2	6.156
SAT1	12.565	13.880	2.487	STAT2	12.442
TNFSF15	4.922	6.236	2.487	TRIM38	7.656
CFLAR	10.140	11.456	2.490	PAPLN	4.771
NEAT1	5.372	6.699	2.508	LGALSL	8.432
DDX58	9.121	10.454	2.519	TP53BP2	9.743
TAB2	8.083	9.416	2.519	NAB1	9.391
IFIT3	7.007	8.341	2.522	IFNGR2	9.511
RAPH1	6.743	8.079	2.524	HCP5	5.213
CFLAR	9.931	11.268	2.526	SNAR-H	15.265
BAMBI	11.368	12.706	2.528	DCP1A	12.419
HIVEP1	9.520	10.860	2.530	SERPINB9	11.714
VNN1	5.524	6.866	2.534	CYTH1	5.773
TRAF2	5.493	6.834	2.534	ANKFY1	6.658
RAB1B	8.356	9.701	2.541	TENT5A	9.890
MAFF	10.037	11.389	2.552		6.053
PTX3	15.839	17.193	2.555	DNAJA1	13.339
SLC2A6	12.191	13.544	2.555	NBAT1	5.228
F2RL1	10.771	12.125	2.557		8.988
	4.670	6.031	2.568	ABCB9	4.862
lnc-OBFC2A-1	5.074	6.435	2.569		4.816
B4GALT1	8.733	10.099	2.578	BCL2L13	5.657
ST5	9.195	10.563	2.581	CDKN1A	8.573
	4.506	5.875	2.582	TMEM62	7.931
TCIM	9.527	10.897	2.583	SAV1	11.990
NINJ1	13.770	15.139	2.583	MED24	9.458
ZNF710	4.876	6.248	2.588		11.357
MOB3C	7.496	8.871	2.595	ACVR1B	4.538
PITPNC1	6.493	7.870	2.597	C5orf15	8.853
SNORD114-28	6.578	7.955	2.597	GNA13	8.719
LOC10272495	6.236	7.616	2.602	SNAR-F	14.294
ITPKC	11.325	12.706	2.604	PNPT1	10.795
LOC10798509	5.486	6.869	2.609	LINC02085	6.843
INSIG1	10.271	11.657	2.612		4.912
LIMK2	11.799	13.188	2.620	HIPK2	7.906
C3orf52	9.241	10.636	2.629	RANBP9	10.020
CAB39	7.919	9.315	2.633	CEMIP2	6.196

MBP	8.393	9.790	2.635	TRANK1	6.509
ARL5B	7.258	8.657	2.637	CD86	6.862
DDX58	9.015	10.419	2.647	PLEKHO2	10.751
PDZD2	6.773	8.179	2.650	PAQR3	5.626
PPP1R2	5.820	7.226	2.651	C19orf12	8.203
CAPN10	5.428	6.835	2.651	BMP2	10.643
VEGFA	6.596	8.006	2.658	SP100	9.289
S1PR2	6.685	8.097	2.662	STAT5A	7.991
LOC100132831	9.533	10.949	2.668	ZFX	4.992
PALLD	9.157	10.573	2.668	LOC107985092	5.486
DUSP16	5.858	7.278	2.677	LOC105369791	5.437
FOSB	5.928	7.350	2.680	C1QTNF1	5.547
C1QTNF1	5.547	6.975	2.690	lnc-MFSD9-4	5.936
BSDC1	6.525	7.957	2.697	PML	12.932
APOL6	9.073	10.506	2.700	HIF1A	7.339
SAMD4A	9.757	11.193	2.706	IL32	11.887
RDX	7.564	9.001	2.707	CD38	7.369
NFKBID	5.013	6.451	2.709	GTPBP2	6.106
RUNX1	8.565	10.006	2.715	RIT1	8.052
IL15RA	9.101	10.546	2.723	MAPKBP1	9.019
SLC30A7	9.288	10.736	2.727		4.723
EHD1	12.763	14.213	2.732	DDX3Y	5.422
TGFB2	6.331	7.781	2.733	NINL	4.836
DTX3L	8.337	9.791	2.741	CAB39	10.558
TRIM56	8.976	10.432	2.743	KLF2	12.265
LOC101929709	6.341	7.798	2.745	SLC30A7	9.288
CEMIP2	6.196	7.659	2.756	PPTC7	5.911
YAE1	10.445	11.908	2.756	DDX3X	10.474
	5.427	6.890	2.758	APH1A	8.234
	4.765	6.230	2.762	LYRM1	9.155
CTSS	5.170	6.640	2.769	RHOB	13.287
SAMD4A	10.817	12.293	2.782	PDE5A	8.911
ZBTB10	5.290	6.768	2.786	CCNL1	8.608
EDNRB	5.405	6.883	2.786	DRAM1	12.915
lnc-THNSL1-2	6.174	7.653	2.788	BDNF-AS	4.467
HELZ2	10.721	12.203	2.793	PLLP	5.284
SRSF6	5.701	7.188	2.803	PSMB8	9.733
AKAP2	7.083	8.572	2.807	SNORA80E	6.691
PMAIP1	10.759	12.251	2.813	SMAD3	11.539
DLEU1-AS1	5.614	7.107	2.814		9.162
MYO1B	5.823	7.318	2.817	LAP3	11.692
C5orf56	10.085	11.591	2.840	lnc-SH2D7-5	5.460
MMP10	13.576	15.084	2.846	LOC101927100	4.929
HIPK1	4.805	6.317	2.850	RUNX1	8.565
SLC41A1	10.555	12.071	2.860	CD40	7.671
GPRC5A	5.120	6.641	2.870	NBN	8.984
MED24	9.458	10.980	2.872	TAP2	11.047
APOL2	12.484	14.006	2.872	PDGFRL	8.164
PAQR3	5.626	7.151	2.878	YAE1	10.445
TRIB1	12.280	13.807	2.883	MCTP1	9.363
NAMPT	12.337	13.870	2.892	HLA-B	11.817
HLA-F	5.873	7.407	2.896	CASP1	9.689
ARID5A	10.508	12.044	2.899	LGALS9	9.593
PGM5	5.043	6.580	2.902	HTATSF1P2	5.869
SLC25A28	8.714	10.253	2.906	PRDM8	7.702
SHB	8.795	10.339	2.916	APH1A	10.295
NAB1	9.069	10.614	2.918	BTG3	13.062

LAMC2	7.347	8.897	2.928	PALLD	11.614
IL15RA	7.466	9.018	2.931	PITPNC1	6.493
TNIP2	9.711	11.269	2.946	LAMC2	7.347
IKBKE	6.088	7.650	2.951	SP100	10.274
RTP4	7.513	9.075	2.953	TNPO3	6.365
NABP1	11.134	12.699	2.958	PTX3	15.839
LGALSL	8.432	10.002	2.969	XLOC_l2_01284	5.641
HIVEP3	6.854	8.426	2.974	IL1RL1	7.367
SLC41A2	5.827	7.402	2.978	UNC93B1	6.218
CAMTA1	4.751	6.331	2.989	KIAA1217	4.974
ARID5A	5.993	7.573	2.989	PLAU	7.588
HRH1	8.045	9.625	2.990	CCNG2	6.453
OVOS2	9.459	11.041	2.995	LOC10192867[illegible]	7.787
GUCY1A1	9.060	10.661	3.034	USP12	5.585
LOC_l2_0032[illegible]	8.234	9.840	3.044	LIMK2	11.799
LAMB3	9.555	11.178	3.080	TNIP2	9.711
UGCG	10.327	11.960	3.101	AZIN2	5.413
IL15RA	4.759	6.394	3.106	PMCHL1	5.356
NRG1	6.788	8.424	3.107	lnc-SMPD2-1	5.674
IL3RA	10.067	11.705	3.113	MMP10	13.576
LINC002481	5.789	7.432	3.123	FUZ	8.302
CHST6	6.128	7.775	3.131	SUSD6	11.720
ARID5B	11.687	13.336	3.135	GUCY1A1	9.060
KCNJ2	8.172	9.822	3.139	LYPD5	5.341
SMCO2	5.778	7.429	3.139	CXCL5	9.058
ZFP36	10.742	12.394	3.142	TNFAIP1	13.551
CEBPD	11.287	12.943	3.151	POU2F2	4.945
FOSL2	8.578	10.246	3.176	CCDC117	10.154
ETS1	7.290	8.958	3.177	NABP1	11.134
TAF4B	4.526	6.195	3.179	NAMPT	12.337
RAPGEF5	5.592	7.270	3.200	BIRC2	11.342
DRD4	4.499	6.184	3.215	USP42	11.127
CXCL11	5.605	7.300	3.238	RNF112	5.509
IFI30	8.306	10.010	3.258	TCIM	9.527
LINC-PINT	4.640	6.344	3.260	NRG1	6.788
NNMT	14.774	16.482	3.267	NR1D1	4.763
SOX7	7.351	9.075	3.305	IL32	10.724
PLPP3	12.732	14.457	3.305	LGALS9	8.157
PLAU	7.588	9.320	3.323	TP53INP2	7.886
NRG1	6.654	8.387	3.324	NR3C1	4.768
TIAM2	11.021	12.758	3.333	ZFPM2	10.196
CYB5R2	7.207	8.949	3.346	NFATC2	5.509
POU2F2	4.945	6.703	3.384	CAMTA1	4.751
CCRL2	8.528	10.291	3.395	CCNL1	8.301
DCUN1D3	11.803	13.567	3.397	GIMAP5	11.856
	5.000	6.768	3.406	lnc-OR4M2-7	7.679
FLJ31104	5.901	7.676	3.423	HRH1	8.045
SPSB1	11.356	13.140	3.444	OR10G2	5.007
TMCC2	6.144	7.928	3.444	WTAP	11.698
[illegible]OC10192912[illegible]	5.475	7.269	3.468	OTUD4	8.092
CREB5	5.479	7.277	3.477	KIAA1217	9.215
PDE5A	8.911	10.715	3.493	FSTL3	9.652
[illegible]OC10050617[illegible]	7.139	8.951	3.511	TRIM26	9.383
APOL4	7.146	8.959	3.514	XBP1	13.480
TICAM1	6.233	8.050	3.523		7.429
GFPT2	7.992	9.810	3.527	S1PR1	11.740
S100A3	8.361	10.180	3.528	ZNF710	4.876

TWIST2	4.888	6.710	3.535	TENT5A	7.879
CNKSR3	6.034	7.857	3.539	RASD1	12.595
SAMD4A	6.247	8.086	3.579	EIF2AK2	7.821
LST1	4.519	6.365	3.595	PARP12	11.048
FSTL3	9.652	11.500	3.599	HLA-A	6.779
	4.912	6.768	3.619	CFLAR	10.146
	5.373	7.229	3.619	LOC100506178	7.139
MAPKBP1	9.019	10.878	3.628	LGALS9	8.193
CNKSR3	12.000	13.864	3.642	BACH1	10.449
RNF19B	6.549	8.420	3.658	ZMYND15	5.406
TNIP1	12.163	14.035	3.660	LOC102724951	6.236
CXCL5	9.058	10.931	3.663		8.068
lnc-IL6-3	6.451	8.326	3.667	CLIC2	9.369
NFKB1	11.364	13.239	3.669	PGM5	5.043
LIMK2	7.376	9.255	3.677	SLC31A2	10.515
PIK3C2B	10.804	12.685	3.684	TNFSF18	5.652
CCRL2	4.938	6.821	3.688	EIF2AK2	11.499
MCTP1	8.087	9.972	3.694	YPEL2	10.304
GUCY1A1	6.556	8.442	3.697	WTAP	8.416
ANO9	5.113	7.016	3.740	N4BP2L1	5.055
LOC101929128	5.663	7.569	3.748	MCL1	10.818
SAT1	7.205	9.114	3.756	MAML2	5.998
CSRNP1	7.042	8.961	3.780	TTC39B	5.814
SIK1	7.192	9.114	3.790	PCDH1	5.890
USP54	9.632	11.562	3.810	MICB-DT	5.817
SBNO2	11.731	13.661	3.810	IL18BP	6.057
AMPD3	7.746	9.680	3.820	NRG1	6.654
ITGB8	6.958	8.897	3.836	VTRNA2-1	4.786
SSH1	9.642	11.587	3.852	lnc-RHOC-1	6.564
PPTC7	5.911	7.858	3.856	RDX	7.564
NABP1	8.503	10.450	3.857	PALLD	9.157
PDLIM4	10.007	11.965	3.886	CTSL	7.559
BCL3	11.887	13.845	3.887	DRD4	4.499
KCNN2	8.772	10.731	3.890	SAMHD1	6.728
IL1RL1	7.367	9.332	3.902	CFLAR	10.140
PFKFB3	5.394	7.361	3.907	IL15RA	6.404
PLA1A	6.492	8.465	3.926	AMPD3	7.746
LOC101927300	6.671	8.664	3.981	LINC-PINT	4.697
LINC01429	6.253	8.261	4.022	DLEU1-AS1	5.614
LOC101928080	4.884	6.894	4.028	PSMB8-AS1	5.918
TNFSF9	7.237	9.264	4.077	PDP1	12.658
ZC3H12C	10.603	12.635	4.092	CA2	10.170
NAMPT	10.444	12.480	4.100	CFLAR	9.931
PITX2	5.036	7.077	4.116	LOC100287049	5.888
CEBPB	11.125	13.167	4.119	TANK	10.696
BCL6	9.301	11.344	4.122	FLT3LG	5.596
IFIH1	8.248	10.296	4.135	HES4	14.042
SLC6A4	10.794	12.861	4.191	PSTPIP2	5.766
ANKRD33B	8.302	10.370	4.193	LOC105371413	6.229
SLC39A14	11.918	13.988	4.200	LOC100507205	4.949
CLDN14	7.656	9.730	4.210	GPRC5A	5.120
MIR155HG	6.240	8.319	4.227	LOC105376287	5.302
MSX1	7.721	9.813	4.263	NINJ1	13.770
LOC100287049	5.888	8.004	4.333	HOXD10	9.465
IL4I1	10.930	13.062	4.382		8.603
PFKFB3	10.628	12.773	4.425	N4BP1	8.934
GBP2	9.514	11.669	4.454	CACNA1A	8.196

NCOA7	12.168	14.343	4.514	SAT1	13.686
WNT5A	5.907	8.083	4.519		4.776
WTAP	8.200	10.408	4.622		5.456
C6orf132	6.820	9.039	4.655	ARL4D	5.283
GRHL1	5.760	7.981	4.662	DDO	5.695
GBP3	9.956	12.187	4.695	B3GNT2	7.493
STX11	5.648	7.885	4.712	MED1	5.918
SPSB1	7.516	9.803	4.880	GRAMD2B	8.951
OLFM1	5.987	8.280	4.903	GTF2B	9.026
lnc-ARRDC3-1	4.570	6.871	4.926	PIK3R3	6.723
TMEM217	8.900	11.204	4.938		4.674
IER3	13.762	16.071	4.954	RNF122	11.058
CSRNP1	5.227	7.562	5.046	SSH1	9.642
LOC10192970[illegible]	4.514	6.858	5.078	SAMD4A	10.817
APOL3	7.645	9.990	5.079		6.491
BDKRB2	8.680	11.029	5.095	REC8	8.084
OLFM1	5.946	8.322	5.192	ERBB2	7.165
IL23A	6.602	8.986	5.219	HLA-J	7.118
RELB	13.336	15.725	5.238	SAMD4A	6.247
FAM129A	6.757	9.168	5.318	LOC646626	9.236
WTAP	4.552	7.011	5.499	SERPINE1	12.318
LOC10050617[illegible]	5.710	8.194	5.596	GATA6	6.047
G0S2	8.811	11.298	5.604	WARS	13.295
LOC10798646[illegible]	5.166	7.655	5.613	GJD3	6.718
BATF3	6.302	8.791	5.614	CASP10	9.167
KCTD8	4.867	7.365	5.648	NLRC5	5.771
lnc-IL6-3	4.612	7.119	5.686	lnc-THNSL1-2	6.174
	8.516	11.040	5.750	SHB	8.795
RASD1	12.595	15.123	5.768	BBC3	12.853
LOC10192970[illegible]	6.232	8.760	5.768	NAB1	9.069
IRAK2	10.408	12.958	5.855	EGR1	10.131
LOC_I2_0002[illegible]	5.168	7.725	5.886	lnc-KBTBD6-1	5.510
CBR3	9.465	12.027	5.903	STAT1	10.306
IER3	11.897	14.463	5.919	FBXO6	6.954
GBP3	8.169	10.751	5.990	KDM7A	5.064
P2RY6	5.339	7.924	6.000	DCP1A	9.858
TNFRSF11B	8.445	11.031	6.003	TRAFD1	12.119
NFKBIA	13.529	16.122	6.033		5.000
CD83	7.755	10.354	6.057	SAMD4A	9.757
MAP3K8	7.619	10.221	6.073	GUCY1A1	6.556
FGF5	5.478	8.089	6.112	TGFB2	6.331
EFNA1	9.494	12.118	6.164	EID3	5.685
RCAN1	11.618	14.283	6.342	NABP1	8.503
CSF1	5.199	7.870	6.372	TICAM1	12.969
ATF3	6.268	8.943	6.390	WNT5A	5.907
POPDC2	6.114	8.798	6.423	RHEBL1	9.355
LINC01767	6.085	8.769	6.426	lnc-IL6-3	6.451
GBP4	5.445	8.131	6.435	LOC10192970[illegible]	6.341
NR4A3	6.112	8.835	6.604	RNF151	5.177
NRG1	8.491	11.230	6.678	ADPRHL2	9.534
IL7R	8.380	11.122	6.690	CYLD	8.064
ATF3	11.021	13.776	6.751	TMEM35A	10.556
NUAK2	9.495	12.253	6.764	FLJ31104	5.901
RRAD	4.645	7.405	6.774	UBQLN1	8.872
SLCO4A1	6.003	8.768	6.801	PDLIM4	10.007
SOCS3	9.035	11.812	6.854	IL3RA	10.067
SP6	7.315	10.119	6.981	B4GALT1	8.733

C1R	7.609	10.437	7.101	PIK3C2B	10.804
TMEM217	9.874	12.715	7.162	BHLHE40	8.042
LOC10192970(	4.557	7.398	7.164	USP54	9.632
GBP5	4.494	7.368	7.329	SIK1	7.192
C1R	5.034	7.920	7.390	ZBTB42	7.826
USP30-AS1	5.520	8.414	7.430	TNFSF9	7.237
FOXF1	5.421	8.347	7.603	MBP	8.393
IL15	4.884	7.811	7.606	CGAS	7.742
CD14	6.484	9.415	7.626	LINC002481	5.789
OXTR	4.649	7.597	7.713	TIAM2	11.021
TLR2	8.686	11.645	7.778	TLE4	5.151
ATF3	4.920	7.900	7.887	PML	6.503
LYPD6	5.114	8.124	8.054	GFPT2	7.992
NFKB2	6.596	9.632	8.202	TMCC2	6.144
JUNB	5.854	8.894	8.226	KLF9	11.167
CCL5	7.199	10.257	8.326	SAT1	12.565
TGFB3	5.299	8.375	8.434	FOSL2	8.578
GBP1	7.856	10.945	8.507	PARP9	6.992
NFKBIZ	7.521	10.618	8.559	BLZF1	9.271
BDKRB1	4.921	8.042	8.700	KCNN2	8.772
ADAMTS4	12.033	15.213	9.062	CEBPD	11.287
CD200	6.716	9.964	9.502	C21orf91	6.442
SDC4	10.123	13.373	9.514	OGFR-AS1	5.173
PTGS2	9.394	12.658	9.606	MCTP1	8.087
NOCT	5.548	8.816	9.634	P2RX7	9.153
RIPK2	11.183	14.464	9.722	TBC1D1	5.915
CITED4	11.731	15.109	10.391	ARL5B	7.258
ICOSLG	7.885	11.279	10.513	TRIM56	8.976
C2CD4B	12.122	15.530	10.611	PLEKHA4	11.050
ADAMTS9	9.488	12.944	10.978	LINC01429	6.253
UBD	12.774	16.242	11.063	lnc-C6orf228-2	5.396
CD200	4.766	8.243	11.130	SIGLEC16	10.295
EBI3	7.643	11.120	11.137	ITGB8	6.958
GCH1	7.069	10.549	11.163	DCP1A	5.814
TNFAIP8	8.803	12.432	12.371	KCNC3	4.749
HIVEP2	8.649	12.369	13.171	TDRD7	10.424
	6.581	10.329	13.435	TWIST2	4.888
ICAM1	10.455	14.209	13.490	ITPKC	11.325
IRF1	7.547	11.313	13.603	CCL3L3	6.736
SLC7A2	10.275	14.070	13.877	TAF4B	4.526
PIM1	6.519	10.321	13.943	RNF213	8.612
ABTB2	4.692	8.514	14.140	SPEM2	4.959
MIR3142HG	7.824	11.663	14.306	GJD3	9.242
ICAM4	5.245	9.103	14.498	lnc-WDR63-1	7.627
TNFAIP8	6.438	10.306	14.598	SNORA27	7.930
LOC10192970(	4.755	8.634	14.712	LOC10192807(	4.907
CCL2	13.596	17.490	14.868	UGCG	10.327
SOD2	11.680	15.580	14.931	LINC00240	5.112
C11orf96	11.207	15.173	15.628		5.466
LTB	8.881	12.854	15.705	C3orf52	9.241
GCH1	5.537	9.532	15.945	DCUN1D3	11.803
TRAF1	7.947	12.221	19.338	PLPP3	12.732
CXCL1	13.338	17.620	19.464		4.765
SERPINA3	6.036	10.333	19.659	DUSP5	8.725
ZC3H12A	6.929	11.387	21.973	KIAA1217	4.913
SELE	11.949	16.539	24.091	LOC10192730(	6.671
TNFAIP2	12.665	17.325	25.277	LIMK2	7.376

CXCL8	9.646	14.407	27.126	LST1	4.519
TNFAIP3	7.488	12.278	27.653	CBX4	5.879
IL18R1	4.826	9.628	27.898	ZBTB10	5.290
BCL2A1	5.032	9.856	28.325	SETD5	5.336
HAS3	4.909	9.881	31.379	OXTR	4.649
VCAM1	8.151	13.247	34.214	NFKB1	11.364
F3	8.444	13.600	35.661	CASP7	11.009
BIRC3	8.821	14.002	36.253	LOC101929128	5.663
MAP3K8	5.191	10.482	39.139	SMCO2	5.778
SOCS1	4.877	10.440	47.287	CCDC71	8.320
RND1	6.502	12.279	54.845	TNFRSF11B	8.445
CXCL1	10.257	16.150	59.447	PANX1	11.041
ACKR3	8.815	14.956	70.550	SLC41A2	5.827
CX3CL1	5.568	11.762	73.167	SLC6A4	10.794
SELE	7.338	13.674	80.801	HIVEP1	9.520
C2CD4A	5.939	12.767	113.641		8.516
CXCL2	8.696	15.592	119.148	BLZF1	5.576
IL6	9.131	16.569	173.373	LOC101929128	5.475
CCL20	6.810	14.539	212.146	ETS1	7.290
CXCL2	7.797	16.553	432.517	LINC-PINT	4.640
CXCL3	4.956	14.716	867.417	NNMT	14.774
				TRIM14	7.573
				CTSS	5.170
				CREB5	5.479
				G0S2	8.811
				SLC35G1	5.063
				DUSP16	5.858
				S100A3	8.361
					6.473
				RNU6ATAC	6.891
					4.686
				LINC00644	6.297
				STC2	6.301
				OLFM1	5.987
				PELI1	11.848
				TTC39A	5.682
				TMEM171	8.400
				IRF2BPL	5.852
				ENTPD5	5.820
				TNIP1	12.163
				JAK2	8.295
				MASTL	6.985
				IL23A	6.602
				MMP25	4.509
				AKAP2	7.083
				SOX7	7.351
				SBNO2	11.731
				P2RX7	9.884
				MAFF	10.037
					6.182
				C6orf132	6.820
				PLSCR1	10.246
				EDNRB	5.405
				SNORA2B	4.956
				HIVEP3	6.854
					12.342
				CCRL2	8.528

FST	9.072
FGF2	6.019
PITX2	5.036
HDX	7.532
UBE2L6	9.044
SAT1	7.205
NAMPT	10.444
PFKFB3	5.394
CNKSR3	12.000
SUSD4	7.940
ZNF107	5.702
PDZD2	6.773
ARID5B	11.687
XLOC_l2_00064	5.224
TRIM14	4.655
TRIM14	7.882
lnc-OBFC2A-1	5.074
OGFR	10.042
lnc-ARRDC3-1	4.570
CLSTN3	11.522
RNF213	5.621
CNKSR3	6.034
FAM129A	6.757
FOSB	5.928
ADAM12	5.790
HOXB9	5.880
LONRF1	5.493
EGR2	4.671
MIR155HG	6.240
CD14	6.484
OLFM1	5.946
ERBB2	4.760
SLC39A14	11.918
LOC10050617[illegible]	5.710
ZC3H7B	10.353
LOC10192970[illegible]	6.232
ZNFX1	4.996
GEM	5.019
lnc-SYNM-2	4.770
IER3	13.762
TICAM1	6.233
APOL1	9.756
NR4A3	6.112
TRIB1	12.280
BCL6	9.301
LOC10192808[illegible]	4.884
NRG1	8.491
THEMIS2	7.685
ACP5	10.051
CLDN14	7.656
POLD1	10.676
ZC3H12C	10.603
IL15RA	9.101
APOBEC3G	6.767
lnc-IL6-3	4.612
S1PR2	6.685
ADAP1	5.980

SPSB1	11.356
PML	8.726
TMEM217	8.900
TNFSF10	10.935
IRF9	8.991
CEBPB	11.125
ADRA2C	5.780
EFNA1	9.494
LOC10041958	6.890
LOC10798646	5.166
CCRL2	4.938
lnc-TOMM20L-	5.610
CLEC4M	6.783
FGF2	7.242
ADAMTS13	7.200
WTAP	4.552
lnc-ROM1-2	5.102
IL15RA	7.466
MAGIX	6.305
ASMTL-AS1	5.320
DDX60L	9.560
TNFSF10	12.154
ANKRD33B	8.302
GRHL1	5.760
IER3	11.897
TRIM14	5.179
HNRNPUL2	7.606
IRAK2	10.408
CD83	7.755
PFKFB3	10.628
RNF213	5.800
IL15RA	4.759
FAM98B	5.324
MPRIP	5.329
SP110	9.373
FZD5	6.438
PSMB9	10.192
PPP4R3A	10.854
LINC01767	6.085
SAMD9L	10.482
NLRC5	4.830
BDKRB2	8.680
BCL3	11.887
WTAP	8.200
KCTD8	4.867
GCA	7.079
ZNFX1	5.147
RELB	13.336
IL4I1	10.930
SLC25A28	8.714
LOC10192970	4.557
PTP4A3	7.249
SPSB1	7.516
ZNF316	6.873
DDX60	7.900
SAMD9L	8.310
ARID5A	10.508

SP6	7.315
ZC3HAV1	11.958
NFKB2	6.596
PARP14	9.579
KIF26A	6.833
CD274	8.603
ARID5A	5.993
CD274	6.674
CITED4	11.731
TRIM21	7.186
NFKBIA	13.529
P2RY6	5.339
RBM20	6.941
GBP3	9.956
OAS3	6.823
ZNFX1	8.186
ATF6B	9.898
ZNF219	10.108
SAMD9	6.508
ISG20	12.929
NFKBIZ	7.521
CBR3	9.465
LYPD6	5.114
C5orf56	10.085
OAS2	8.148
LOC10192970	4.514
GBP2	9.514
PLEKHF1	5.421
DHX58	9.149
DTX3L	8.337
MSX1	7.721
	5.546
ZFP36	10.742
RRAD	4.645
MAP3K8	7.619
TMEM217	9.874
MOB3C	7.496
TLR2	8.686
PARP9	7.388
APOL4	7.146
POPDC2	6.114
GM2A	8.499
lnc-FAM184B-1	4.509
IFI35	8.849
KLHL35	6.553
MIR3142HG	7.824
RCAN1	11.618
CSF1	5.199
TAP1	12.327
CSRNP1	7.042
ICOSLG	7.885
NCOA7	12.168
IL7R	8.380
IFI44	10.227
EBI3	7.643
LOC10537418	7.447
XLOC_l2_0058	6.328

TGFB3	5.299
APOL2	12.484
HLA-F	5.873
SBNO2	12.004
TMEM229B	5.134
FGF5	5.478
USP18	10.013
PPM1K	6.004
RNF19B	6.549
STX11	5.648
ASPHD1	7.110
IRF7	10.405
PIM1	6.519
ABTB2	4.692
OAS1	8.319
SERPINA3	6.036
SLCO4A1	6.003
PLA1A	6.492
BST2	6.814
ADAMTS9	9.488
LGALS7	5.207
SDC4	10.123
BST2	7.511
FZD9	7.227
APOL3	7.645
CD200	6.716
CTSZ	12.266
CD200	4.766
IFI6	9.760
FAM83H-AS1	7.048
CSRNP1	5.227
	6.581
LTB	8.881
TNFAIP8	8.803
USP41	6.453
C2CD4B	12.122
PTGS2	9.394
BDKRB1	4.921
RARRES3	8.406
PMAIP1	10.759
SOCS3	9.035
SLC7A2	10.275
USP30-AS1	5.520
RIPK2	11.183
RAPGEF5	5.592
C11orf96	11.207
lnc-POLD3-2	4.954
IFI30	8.306
BATF3	6.302
TNFAIP8	6.438
C1R	7.609
FOXF1	5.421
JUNB	5.854
ADAMTS4	12.033
INSYN1	7.660
ICAM1	10.455
ZC3H12A	6.929

UBD	12.774
C1R	5.034
NUAK2	9.495
RALGDS	10.562
SLC15A3	7.448
ZC3HAV1	7.278
GBP3	8.169
IFIT5	9.783
HELZ2	10.721
HAS3	4.909
SOD2	11.680
HIVEP2	8.649
APOL6	9.073
CCL2	13.596
LOC10192970[illegible]	4.755
CXCL1	13.338
BCL2A1	5.032
NOCT	5.548
	5.794
TRAF1	7.947
IL18R1	4.826
SELE	11.949
ISG15	12.260
GCH1	7.069
ICAM4	5.245
DDX58	9.121
ATF3	11.021
IL15	4.884
GCH1	5.537
CXCL8	9.646
DDX58	9.015
ATF3	6.268
XLOC_l2_0002[illegible]	5.168
TNFAIP2	12.665
STAC3	6.187
LOC10050754[illegible]	7.725
IRF1	7.547
ATF3	4.920
TNFAIP3	7.488
GBP1	7.856
F3	8.444
VCAM1	8.151
OAS2	5.062
SECTM1	6.651
MAP3K8	5.191
LOC10537988[illegible]	6.366
IFIH1	8.248
BIRC3	8.821
CXCL1	10.257
MX1	9.984
	6.358
	6.304
PKP4-AS1	4.765
IFITM1	5.418
GBP5	4.494
RTP4	7.513
NUDT13	4.899

UBE2I	4.962
ACKR3	8.815
TNXB	7.026
SELE	7.338
XLOC_l2_01346	7.262
RND1	6.502
CXCL2	8.696
CX3CL1	5.568
SOCS1	4.877
C2CD4A	5.939
lnc-SLC43A1-1	5.402
ADGRB2	7.244
CCL5	7.199
lnc-GIP-1	6.432
DUSP9	5.697
GBP4	5.445
LOC105370941	5.016
MAGEE1	7.041
CMPK2	5.797
CCL20	6.810
IL6	9.131
XLOC_l2_00683	4.669
	5.959
IFIT3	7.007
	5.318
CXCL11	5.605
CXCL2	7.797
MIR4432HG	5.138
CXCL3	4.956
IFIT2	6.909
IFIT1	6.362

ed with LPS (10 ng/ml) or poly (I:C) (100μg/ml) for 4 hours.

level (log2)

poly (I:C)	fold change
6.732	-8.912
6.047	-8.197
5.695	-8.043
6.646	-7.042
6.325	-6.856
5.028	-6.810
6.459	-6.810
9.131	-6.807
5.023	-6.640
10.152	-6.126
7.388	-6.121
7.392	-5.587
8.806	-5.457
6.133	-5.433
6.489	-5.178
8.846	-5.161
11.061	-5.138
10.303	-4.920
9.828	-4.908
8.329	-4.834
11.535	-4.766
12.141	-4.739
7.509	-4.577
11.394	-4.552
8.408	-4.494
12.943	-4.491
11.048	-4.418
7.891	-4.400
4.867	-4.268
10.155	-4.233
12.647	-4.159
4.910	-4.157
9.520	-4.153
9.568	-4.062
7.189	-4.036
10.418	-4.021
7.545	-4.001
8.707	-3.963
7.038	-3.916
7.500	-3.895
8.188	-3.890
12.255	-3.815
5.461	-3.810
10.216	-3.798
8.018	-3.795
11.602	-3.721
11.553	-3.720
13.070	-3.703
4.511	-3.694
12.668	-3.649
12.249	-3.643
10.222	-3.593
6.512	-3.574

10.048	-3.508
5.329	-3.495
12.156	-3.488
9.233	-3.487
8.095	-3.476
9.162	-3.472
7.011	-3.469
5.324	-3.459
4.606	-3.398
8.032	-3.381
5.043	-3.363
8.953	-3.362
4.980	-3.361
8.680	-3.346
10.155	-3.325
6.876	-3.318
5.308	-3.317
4.520	-3.303
5.764	-3.294
8.451	-3.291
5.633	-3.280
11.394	-3.273
8.164	-3.250
5.519	-3.230
10.266	-3.222
11.133	-3.219
8.206	-3.214
5.184	-3.208
11.694	-3.140
6.420	-3.136
8.056	-3.133
11.054	-3.126
9.480	-3.120
6.821	-3.115
4.944	-3.109
6.553	-3.101
9.155	-3.099
10.111	-3.084
5.353	-3.074
5.613	-3.062
8.371	-3.058
7.010	-3.042
11.755	-3.031
6.267	-3.028
13.169	-3.026
8.976	-2.988
6.119	-2.982
15.139	-2.973
8.601	-2.952
9.189	-2.946
4.670	-2.942
5.731	-2.940
6.318	-2.931
7.531	-2.926
5.605	-2.919
7.809	-2.918
6.303	-2.915

6.331	-2.905
4.673	-2.902
6.404	-2.894
7.445	-2.888
4.812	-2.885
10.534	-2.871
8.194	-2.863
10.932	-2.857
11.207	-2.846
5.596	-2.835
6.029	-2.835
6.501	-2.825
7.012	-2.821
12.838	-2.821
5.107	-2.819
8.171	-2.805
12.853	-2.787
9.435	-2.785
7.591	-2.776
9.937	-2.765
9.769	-2.764
9.606	-2.760
12.941	-2.755
9.376	-2.753
10.025	-2.750
12.589	-2.747
7.572	-2.741
9.107	-2.739
10.975	-2.739
7.376	-2.737
5.126	-2.732
9.782	-2.725
12.370	-2.723
13.717	-2.723
8.774	-2.719
8.713	-2.717
5.333	-2.717
9.233	-2.715
7.645	-2.713
8.917	-2.708
14.103	-2.705
10.483	-2.701
7.157	-2.701
8.455	-2.688
10.817	-2.686
5.119	-2.674
8.442	-2.665
9.867	-2.654
7.295	-2.648
5.465	-2.647
4.781	-2.644
5.696	-2.638
4.884	-2.630
5.168	-2.628
8.025	-2.611
10.136	-2.606
13.336	-2.604

8.615	-2.603
11.561	-2.603
8.857	-2.598
5.801	-2.568
9.075	-2.567
8.161	-2.563
6.562	-2.559
9.228	-2.559
4.834	-2.556
9.377	-2.552
6.131	-2.546
6.397	-2.542
6.579	-2.533
8.373	-2.532
4.915	-2.529
4.923	-2.528
4.816	-2.528
9.976	-2.527
14.612	-2.521
10.901	-2.521
13.017	-2.517
9.011	-2.515
6.026	-2.512
7.113	-2.510
6.151	-2.503
12.655	-2.503
11.954	-2.496
6.956	-2.494
6.727	-2.470
14.714	-2.468
8.054	-2.466
5.064	-2.465
10.065	-2.462
8.214	-2.461
5.747	-2.460
10.863	-2.457
4.942	-2.456
6.761	-2.454
6.502	-2.450
5.080	-2.450
7.179	-2.447
5.090	-2.437
10.030	-2.436
4.965	-2.436
9.381	-2.435
7.727	-2.431
4.902	-2.425
7.415	-2.423
11.297	-2.418
5.098	-2.412
9.021	-2.411
12.267	-2.411
11.873	-2.406
7.306	-2.406
9.797	-2.405
8.444	-2.404
7.853	-2.403

9.472	-2.386
9.139	-2.381
12.116	-2.378
11.143	-2.376
8.387	-2.371
6.458	-2.366
5.426	-2.365
8.987	-2.363
13.398	-2.361
7.160	-2.360
8.188	-2.354
11.193	-2.354
7.002	-2.349
8.157	-2.349
10.893	-2.347
7.679	-2.346
6.060	-2.345
6.367	-2.342
6.699	-2.341
5.812	-2.338
8.597	-2.335
5.944	-2.331
10.605	-2.331
6.744	-2.322
5.409	-2.321
12.615	-2.320
9.394	-2.319
8.001	-2.314
9.499	-2.310
7.292	-2.309
4.614	-2.305
11.411	-2.304
7.744	-2.301
4.788	-2.300
10.047	-2.298
12.867	-2.296
8.070	-2.296
4.758	-2.294
8.826	-2.293
6.169	-2.293
5.841	-2.292
5.499	-2.292
4.880	-2.290
7.841	-2.290
7.012	-2.289
6.268	-2.288
8.882	-2.283
9.478	-2.280
4.649	-2.279
9.682	-2.276
13.751	-2.275
5.906	-2.275
10.913	-2.273
8.424	-2.272
6.006	-2.266
5.384	-2.265
5.706	-2.262

5.699	-2.259
10.611	-2.256
8.528	-2.253
5.623	-2.248
7.774	-2.248
9.064	-2.248
11.545	-2.247
8.382	-2.244
5.096	-2.244
5.661	-2.243
12.000	-2.242
8.246	-2.242
5.956	-2.241
8.135	-2.241
15.261	-2.241
4.872	-2.239
4.988	-2.236
10.283	-2.236
4.843	-2.236
5.768	-2.233
7.629	-2.227
5.275	-2.226
12.117	-2.221
5.953	-2.219
4.864	-2.217
6.528	-2.216
5.906	-2.216
5.152	-2.210
8.031	-2.209
5.233	-2.209
9.885	-2.208
5.140	-2.204
13.395	-2.204
6.018	-2.199
9.688	-2.198
7.227	-2.196
5.902	-2.193
4.715	-2.189
4.726	-2.187
11.910	-2.179
8.409	-2.179
6.138	-2.178
10.599	-2.176
12.745	-2.173
7.815	-2.171
5.641	-2.168
8.600	-2.167
4.671	-2.167
5.222	-2.165
7.929	-2.163
7.080	-2.162
9.766	-2.157
10.889	-2.157
6.415	-2.156
13.147	-2.155
9.029	-2.155
14.298	-2.154

7.735	-2.154
6.244	-2.150
5.744	-2.150
8.537	-2.149
7.074	-2.148
5.549	-2.145
6.436	-2.143
5.609	-2.141
11.100	-2.140
4.908	-2.139
7.108	-2.138
11.466	-2.138
6.433	-2.138
7.101	-2.135
13.336	-2.133
9.211	-2.129
5.238	-2.129
6.083	-2.128
9.538	-2.125
7.203	-2.123
6.423	-2.123
9.453	-2.122
13.921	-2.121
7.556	-2.121
5.606	-2.118
8.996	-2.117
7.584	-2.117
7.234	-2.112
8.759	-2.110
4.720	-2.109
5.512	-2.108
5.914	-2.108
12.292	-2.106
5.177	-2.104
5.663	-2.104
10.192	-2.104
5.133	-2.102
6.298	-2.101
8.796	-2.097
6.570	-2.096
7.884	-2.096
9.835	-2.095
7.575	-2.094
5.734	-2.093
4.798	-2.092
10.862	-2.090
9.610	-2.089
6.532	-2.088
9.687	-2.087
4.798	-2.087
7.735	-2.086
5.186	-2.085
5.550	-2.084
6.002	-2.081
8.284	-2.081
8.567	-2.079
4.717	-2.077

4.526	-2.075
7.549	-2.075
5.357	-2.075
11.504	-2.073
4.754	-2.072
4.361	-2.071
9.469	-2.070
7.166	-2.069
9.501	-2.068
5.392	-2.067
5.278	-2.060
8.507	-2.059
8.880	-2.059
10.263	-2.059
8.024	-2.059
7.515	-2.057
9.574	-2.056
7.820	-2.055
9.273	-2.054
10.586	-2.052
6.487	-2.052
5.269	-2.051
6.240	-2.048
9.595	-2.047
5.511	-2.047
10.257	-2.047
7.538	-2.046
8.131	-2.044
4.918	-2.041
9.083	-2.040
9.901	-2.039
4.867	-2.037
7.817	-2.035
9.015	-2.035
12.456	-2.035
9.915	-2.035
4.396	-2.034
8.124	-2.034
6.203	-2.033
5.861	-2.033
6.834	-2.031
5.790	-2.028
10.249	-2.028
6.921	-2.027
8.483	-2.024
5.320	-2.023
10.909	-2.022
5.444	-2.021
5.550	-2.021
14.598	-2.020
4.443	-2.020
6.248	-2.020
5.806	-2.020
8.866	-2.014
6.109	-2.012
6.313	-2.011
4.565	-2.011

4.669	-2.010
7.279	-2.010
5.027	-2.009
15.069	-2.009
4.708	-2.008
5.554	-2.007
11.027	-2.007
5.224	-2.006
5.148	-2.005
5.975	-2.005
9.620	-2.004
13.539	-2.002
9.530	-2.002
7.468	-2.001
8.848	-2.001
7.860	-2.001
9.077	-2.000
5.379	-2.000
6.078	2.000
10.949	2.001
9.823	2.001
7.615	2.002
15.946	2.004
10.723	2.006
14.091	2.006
9.380	2.007
12.691	2.007
17.822	2.010
8.445	2.011
11.140	2.013
7.797	2.015
13.099	2.016
6.526	2.018
9.092	2.024
16.117	2.028
10.816	2.029
6.844	2.029
7.425	2.030
6.162	2.033
16.940	2.035
6.645	2.035
6.409	2.036
6.091	2.037
10.528	2.037
6.571	2.038
6.122	2.038
13.620	2.040
7.840	2.043
6.154	2.043
11.133	2.043
10.862	2.047
9.901	2.049
7.999	2.055
10.126	2.061
8.361	2.061
5.976	2.063
5.652	2.064

8.966	2.065
5.888	2.067
9.845	2.067
6.646	2.068
8.256	2.069
9.341	2.073
7.677	2.079
12.300	2.081
8.281	2.081
7.146	2.082
12.020	2.083
8.273	2.084
7.188	2.085
9.294	2.086
10.519	2.086
6.181	2.089
11.836	2.092
12.274	2.093
7.384	2.094
6.425	2.096
5.873	2.096
10.108	2.099
6.372	2.101
9.353	2.101
16.129	2.102
14.919	2.102
9.850	2.103
11.837	2.110
12.179	2.113
15.423	2.114
6.260	2.115
10.165	2.116
10.511	2.116
5.715	2.116
9.922	2.116
12.763	2.118
5.931	2.119
8.998	2.120
7.079	2.120
8.104	2.124
13.852	2.128
8.704	2.129
8.231	2.131
10.445	2.131
10.868	2.132
14.350	2.133
9.917	2.133
12.078	2.137
6.635	2.143
12.398	2.145
6.931	2.146
6.570	2.147
6.476	2.148
17.391	2.149
13.014	2.150
5.879	2.150
10.638	2.151

9.220	2.151
12.159	2.152
10.671	2.155
11.924	2.157
14.441	2.159
11.666	2.160
7.154	2.164
6.947	2.165
11.662	2.165
9.034	2.167
10.025	2.171
10.958	2.173
10.137	2.173
11.946	2.174
6.102	2.176
8.574	2.178
10.679	2.180
6.075	2.182
6.825	2.185
5.889	2.186
7.285	2.186
13.571	2.187
8.785	2.188
5.904	2.192
9.565	2.193
10.877	2.194
10.525	2.195
10.646	2.195
6.348	2.196
16.400	2.197
13.555	2.198
12.850	2.198
6.911	2.201
7.798	2.203
11.030	2.204
7.193	2.204
14.480	2.205
6.370	2.206
10.129	2.206
6.007	2.210
5.964	2.217
6.805	2.217
9.722	2.218
9.082	2.220
13.141	2.221
10.610	2.221
12.510	2.224
5.691	2.224
10.007	2.225
9.874	2.226
15.449	2.227
11.951	2.227
7.999	2.229
6.069	2.229
9.063	2.230
11.178	2.232
7.355	2.233

7.668	2.234
8.027	2.242
11.917	2.243
6.795	2.248
9.372	2.248
11.812	2.249
10.462	2.254
9.164	2.254
6.165	2.255
6.659	2.255
6.611	2.256
6.724	2.261
7.113	2.262
14.114	2.268
8.523	2.271
13.071	2.273
8.557	2.278
7.294	2.278
9.241	2.280
10.210	2.283
5.915	2.285
6.614	2.285
6.028	2.285
11.751	2.286
13.459	2.288
10.484	2.290
7.108	2.292
11.671	2.292
9.432	2.294
10.354	2.295
14.488	2.299
10.112	2.299
9.811	2.302
14.120	2.306
5.672	2.306
6.490	2.307
10.940	2.309
7.903	2.317
12.759	2.330
10.383	2.332
12.914	2.332
6.686	2.340
6.155	2.340
9.792	2.340
8.904	2.351
10.218	2.351
12.281	2.351
9.398	2.352
11.680	2.353
10.598	2.354
13.053	2.356
10.929	2.362
10.833	2.363
7.110	2.363
8.943	2.364
11.537	2.366
14.304	2.366

12.858	2.369
7.738	2.370
8.593	2.371
11.519	2.371
7.612	2.373
17.087	2.375
6.890	2.377
8.617	2.378
7.470	2.382
6.229	2.386
8.843	2.387
7.708	2.387
9.042	2.388
6.842	2.389
13.057	2.392
10.970	2.393
6.674	2.396
6.617	2.397
6.937	2.399
14.839	2.400
9.566	2.402
12.985	2.403
10.325	2.403
6.608	2.406
10.327	2.411
14.821	2.412
6.217	2.415
11.429	2.419
12.409	2.419
13.613	2.422
12.619	2.423
12.405	2.425
6.788	2.426
10.807	2.428
8.068	2.429
6.044	2.430
12.007	2.432
9.442	2.437
9.173	2.439
6.057	2.444
11.490	2.451
6.803	2.452
6.046	2.453
9.598	2.457
13.154	2.457
8.979	2.462
9.347	2.465
6.310	2.468
13.002	2.469
9.399	2.474
10.522	2.475
10.961	2.477
10.695	2.484
14.793	2.484
8.744	2.487
13.057	2.491
6.197	2.498

9.201	2.500
13.918	2.503
9.164	2.536
12.394	2.540
8.125	2.541
11.493	2.544
8.488	2.549
9.543	2.549
11.799	2.550
6.757	2.551
7.588	2.553
9.421	2.553
10.722	2.555
6.397	2.557
11.869	2.557
7.008	2.560
12.855	2.560
11.668	2.574
9.781	2.576
6.420	2.577
12.184	2.578
7.367	2.583
7.186	2.588
7.262	2.589
7.190	2.591
7.432	2.594
8.030	2.595
6.170	2.609
7.950	2.613
8.956	2.626
10.556	2.637
8.959	2.640
5.901	2.643
8.131	2.645
11.546	2.650
7.811	2.652
9.155	2.656
6.109	2.661
7.028	2.664
7.333	2.666
14.074	2.669
11.587	2.670
11.353	2.679
7.313	2.685
12.121	2.686
7.022	2.686
15.468	2.687
7.193	2.690
7.658	2.693
6.379	2.696
6.551	2.696
6.735	2.700
15.203	2.700
10.902	2.707
10.040	2.707
10.372	2.710
9.634	2.711

15.127	2.714
6.218	2.716
6.898	2.717
6.725	2.718
7.143	2.728
8.943	2.731
7.369	2.735
10.402	2.735
10.483	2.744
8.180	2.747
6.136	2.756
12.523	2.761
11.109	2.765
12.285	2.766
7.961	2.769
9.568	2.796
8.652	2.804
8.607	2.807
7.739	2.814
10.730	2.816
13.814	2.821
7.547	2.828
14.798	2.834
8.226	2.844
10.677	2.849
7.286	2.859
7.690	2.860
10.312	2.863
14.374	2.870
10.592	2.873
11.653	2.873
7.034	2.877
11.832	2.879
8.482	2.884
6.593	2.888
11.391	2.894
13.654	2.898
6.539	2.906
11.300	2.916
8.100	2.916
7.876	2.919
7.233	2.924
10.051	2.925
14.523	2.935
7.462	2.937
10.910	2.939
8.007	2.941
7.900	2.946
6.737	2.947
11.095	2.952
9.626	2.953
12.119	2.955
7.464	2.956
10.439	2.962
11.575	2.965
11.635	2.966
10.301	2.966

12.376	2.972
9.625	2.996
11.217	3.000
8.778	3.002
9.420	3.020
8.832	3.020
9.989	3.025
9.343	3.033
7.391	3.035
12.623	3.036
6.756	3.040
8.112	3.051
9.602	3.053
7.760	3.065
12.786	3.070
14.184	3.071
10.207	3.093
8.626	3.104
10.908	3.110
10.409	3.111
12.929	3.121
8.086	3.126
6.818	3.126
9.732	3.126
10.803	3.137
7.565	3.140
8.911	3.145
10.631	3.150
12.708	3.156
7.913	3.161
7.058	3.165
11.959	3.168
8.623	3.172
7.480	3.174
6.417	3.178
12.094	3.181
6.561	3.188
13.006	3.206
8.418	3.209
6.208	3.209
10.308	3.239
6.657	3.242
10.949	3.264
9.348	3.297
9.652	3.300
6.632	3.304
12.053	3.308
6.844	3.321
7.198	3.322
10.977	3.331
13.541	3.334
14.476	3.350
6.512	3.356
10.473	3.358
6.662	3.361
8.420	3.362
9.127	3.366

6.271	3.369
7.632	3.371
7.046	3.378
7.093	3.380
6.408	3.384
13.124	3.388
12.770	3.388
7.426	3.394
7.545	3.403
10.091	3.413
10.222	3.428
12.826	3.445
7.612	3.446
12.582	3.454
11.312	3.462
10.308	3.464
7.370	3.468
7.269	3.468
9.088	3.477
6.447	3.501
16.586	3.510
9.387	3.517
6.987	3.522
7.298	3.527
10.636	3.542
6.897	3.564
7.698	3.581
10.216	3.616
8.328	3.619
8.750	3.627
6.546	3.630
8.160	3.637
8.165	3.639
7.854	3.650
13.716	3.651
7.551	3.654
10.273	3.664
7.725	3.664
7.701	3.682
14.045	3.686
10.178	3.688
8.877	3.711
8.495	3.713
6.406	3.724
8.986	3.738
9.254	3.739
13.640	3.756
11.794	3.757
11.950	3.764
8.097	3.772
8.735	3.772
12.165	3.783
7.328	3.792
6.880	3.794
8.784	3.810
14.273	3.813
10.459	3.814

11.020	3.859
7.973	3.875
6.997	3.894
9.496	3.901
11.011	3.909
9.178	3.926
12.419	3.931
7.373	3.941
13.979	3.943
9.926	3.961
7.695	3.979
8.769	3.988
13.691	4.011
7.229	4.012
6.663	4.022
9.892	4.026
7.088	4.040
12.060	4.049
6.590	4.054
13.549	4.076
7.651	4.085
8.067	4.093
8.793	4.099
7.964	4.102
7.826	4.104
7.918	4.107
7.532	4.109
6.731	4.169
8.311	4.203
8.558	4.210
8.020	4.211
6.838	4.223
14.000	4.233
7.800	4.257
12.470	4.336
8.350	4.341
7.114	4.342
7.139	4.347
6.893	4.354
15.886	4.359
8.359	4.366
11.884	4.370
8.253	4.411
14.437	4.460
11.469	4.496
7.054	4.498
10.660	4.499
9.867	4.538
12.237	4.549
9.843	4.553
12.864	4.556
12.791	4.558
11.291	4.562
8.967	4.594
6.812	4.597
8.888	4.607
8.205	4.674

13.581	4.674
10.965	4.720
11.142	4.732
13.194	4.784
11.251	4.791
13.389	4.803
8.054	4.837
11.774	4.859
9.173	4.866
7.453	4.882
7.226	4.883
7.900	4.891
9.075	4.898
9.536	4.904
9.498	4.919
6.857	4.941
7.407	4.943
9.773	4.948
8.617	4.965
7.635	4.975
11.876	4.979
14.473	4.990
10.629	5.015
8.087	5.018
14.227	5.027
7.519	5.063
9.953	5.085
12.755	5.088
10.104	5.095
12.989	5.137
8.168	5.164
7.133	5.185
7.713	5.235
7.717	5.236
11.762	5.238
8.849	5.319
12.607	5.333
13.270	5.337
8.518	5.400
12.917	5.409
7.272	5.435
11.162	5.584
14.376	5.613
10.690	5.619
7.380	5.709
9.606	5.764
7.679	5.785
15.873	5.802
13.470	5.816
11.259	5.834
7.105	5.847
9.804	5.879
10.077	5.898
9.442	5.936
10.472	5.945
10.885	5.960
13.087	5.976

9.916	6.065
14.564	6.091
9.213	6.138
12.209	6.189
9.468	6.212
11.242	6.226
8.650	6.309
9.333	6.313
14.394	6.334
9.853	6.352
16.204	6.388
8.019	6.407
9.647	6.524
12.682	6.620
9.552	6.631
10.927	6.685
12.656	6.765
12.873	6.797
9.276	6.814
15.702	6.834
10.296	6.844
12.244	6.863
7.897	6.882
12.871	6.901
10.936	6.905
7.306	6.928
12.310	6.946
8.235	7.034
11.963	7.035
11.158	7.071
10.543	7.071
8.371	7.088
13.573	7.118
7.481	7.138
10.466	7.194
12.729	7.232
10.353	7.246
11.544	7.251
10.258	7.310
10.019	7.325
8.999	7.383
11.414	7.542
7.442	7.642
11.796	7.712
9.506	7.742
10.780	7.760
14.577	7.775
8.158	7.776
15.306	7.884
10.027	7.919
10.876	7.954
15.172	8.020
11.392	8.069
13.248	8.117
10.669	8.143
10.476	8.159
9.356	8.160

8.331	8.179
15.539	8.308
8.951	8.443
15.083	8.451
8.222	8.502
8.568	8.518
13.104	8.519
9.096	8.526
9.643	8.542
8.767	8.684
10.242	8.764
13.569	8.966
9.686	8.977
7.861	8.990
11.497	9.047
9.219	9.078
9.190	9.107
9.711	9.313
10.035	9.321
12.716	9.372
8.455	9.505
13.375	9.524
10.772	9.584
10.493	9.620
10.912	9.622
10.004	9.764
15.560	9.811
8.062	9.821
13.061	9.854
10.371	10.010
8.550	10.012
9.925	10.152
12.240	10.264
12.201	10.542
9.869	10.677
15.547	10.738
12.869	11.120
8.400	11.151
11.895	11.227
14.252	11.255
12.534	11.302
13.778	11.335
9.028	11.379
14.705	11.492
9.141	11.705
14.787	11.959
8.536	11.975
11.896	12.046
9.901	12.112
10.049	12.223
11.231	12.316
9.046	12.340
9.487	12.413
15.683	12.551
11.330	12.727
14.130	12.769
10.614	12.863

16.480	13.047
8.831	13.894
13.294	13.921
14.369	13.994
11.283	14.274
11.122	14.361
12.013	14.368
13.633	14.425
14.626	14.976
8.821	15.050
15.624	15.389
12.594	15.403
13.044	15.682
17.606	16.118
8.783	16.308
17.433	17.096
9.138	17.210
9.665	17.348
9.986	18.278
12.141	18.295
9.072	18.984
16.274	20.045
16.600	20.247
11.423	20.460
9.626	20.840
13.504	20.855
15.405	20.871
9.274	20.964
9.994	21.968
14.131	22.392
13.529	22.845
10.818	23.434
9.731	23.641
17.268	24.309
10.899	26.214
12.447	26.405
12.504	31.050
9.891	31.362
12.520	32.719
12.972	34.660
13.567	34.856
13.305	35.601
10.224	35.813
11.840	36.474
10.465	38.687
11.658	39.167
13.618	41.364
14.221	42.202
15.669	42.591
15.402	42.752
11.847	44.907
11.952	50.120
10.450	51.464
11.145	52.983
10.249	53.999
13.294	54.976
10.763	58.246

10.835	58.580
14.883	67.088
13.141	69.298
13.462	69.748
13.496	75.274
12.757	76.413
15.129	86.389
12.107	92.970
11.425	93.584
12.490	93.800
12.043	99.769
13.904	101.137
13.950	107.726
13.258	113.426
12.526	113.676
12.379	122.203
12.056	131.671
14.137	136.766
13.012	148.633
14.030	149.091
16.482	163.225
12.596	243.373
13.904	246.407
15.174	287.474
13.487	287.873
13.910	316.309
16.106	317.134
13.552	340.948
13.670	419.933
15.729	451.858
15.808	697.383
